# Exploring the Role of Meaning in Life in Relation to Burn-Out Symptoms Among Early and Mid-Career Nurses: A Cross-Sectional Study

**DOI:** 10.1155/jonm/1205579

**Published:** 2025-11-25

**Authors:** Hendrik Van Simaeys, Els Clays, Lilian Lechner, Emelien Lauwerier

**Affiliations:** ^1^Department of Public Health and Primary Care, Faculty of Medicine and Health Sciences, Ghent University, Ghent, Belgium; ^2^Department of Health Psychology, Faculty of Psychology, Open University, Heerlen, the Netherlands

**Keywords:** burnout, job demands-resources model, meaning in life, nurses, occupational health, psychosocial resources

## Abstract

**Background:**

Burnout negatively affects nurses' well-being and patient care. This study examines whether meaning in life, conceptualized as presence (sense that life is meaningful) and search (active pursuit of meaning), is associated with burnout symptoms among early- and mid-career nurses. Additionally, we explore whether job resources (influence at work, supervisor and colleague support, and professional development) moderate these associations.

**Methods:**

A cross-sectional analysis was conducted using baseline data from a longitudinal panel study among 375 nurses (≤ 10 years of experience) across six hospitals in Flanders, Belgium. Data were collected via an online survey in March-April 2024. Burnout symptoms were assessed using the Burnout Assessment Tool, and meaning in life using the Meaning in Life Questionnaire. Job resources, including supervisor and colleague support, influence at work, and professional development, were measured using the Copenhagen Psychosocial Questionnaire (COPSOQ) and tested as moderators. Demographic and occupational variables were included as covariates. Pearson correlations and multiple linear regressions were performed.

**Findings:**

Presence of meaning was negatively associated with burnout symptoms (*B* = −0.037, *p* < 0.001), whereas search for meaning was positively associated with burnout (*B* = 0.011, *p* < 0.05). Job resources moderated these associations. The negative association between presence of meaning and symptoms such as exhaustion and emotional dysregulation was stronger in high-resource contexts. In contrast, the positive association between search for meaning and symptoms like exhaustion and mental distancing was only observed in low-resource contexts.

**Discussion:**

Presence and search for meaning relate differently to burnout symptoms. Presence was linked to fewer symptoms, while search showed a positive association, especially in low-resource settings. Strengthening job resources and supporting a stable sense of meaning, rather than prolonged search, may contribute to nurses' well-being. Targeted initiatives such as mentorship and career development could help foster resilience and meaningful work engagement.

## 1. Introduction

Research highlights an increased risk of mental health issues such as burnout among healthcare professionals [1]. The emotionally intensive nature of healthcare work, coupled with systemic challenges like staff shortages and administrative burdens, contributes to chronic stress and psychological exhaustion [2]. Among healthcare workers, nurses are particularly susceptible to burnout due to their sustained patient interactions, which often involve significant emotional and cognitive demands [3]. Burnout can be characterized by exhaustion, mental distance, and cognitive and emotional dysregulation [4]. It has been widely documented among nurses and is associated with reduced professional efficacy, increased medical errors, and diminished quality of care [5]. Early- and mid-career nurses face an elevated risk as they transition from education to professional practice, adapt to workplace realities, and develop their professional identity [6–8]. These challenges make it crucial to identify protective factors that mitigate burnout risk.

One such factor is meaning in life, a key determinant of psychological well-being and resilience [9]. It comprises coherence (the sense that life is comprehensible), purpose (a sense of direction and goals), and significance (the belief that life has inherent value) [10]. Research distinguishes between presence of meaning (the extent to which individuals experience life as meaningful) and search for meaning (the active pursuit of meaning and purpose) [11]. While presence of meaning provides stability and helps individuals navigate stressors, search for meaning is a more dynamic process that, although sometimes accompanied by discomfort, fosters identity formation and aligns personal values with external challenges. In occupational contexts, work is a key source through which individuals experience and derive meaning, particularly in professions such as nursing where caregiving and identity are closely intertwined [12]. Broader meaning in life provides an overarching framework within which the meaning of work can be situated and interpreted. However, when work environments become challenging and stress accumulates, the resulting strain or burnout often spills over into other areas of personal life [13]. This underscores the importance of situating meaning in life within occupational contexts when studying occupational well-being. When individuals experience their broader life as meaningful, they may interpret job demands as more purposeful and manageable. Conversely, when meaning in life is threatened, occupational stressors may become more overwhelming, amplifying burnout symptoms [12, 13].

In high-stress professions such as nursing, meaning in life supports psychological stability and adaptive coping mechanisms, potentially mitigating burnout risk [14, 15]. Recent research among teachers suggests that meaning in life is associated with lower occupational stress by offering a framework for processing workplace challenges [16]. Similarly, in hospice nurses, the presence of meaning in life has been associated with greater resilience, enabling them to endure the emotional strain of prolonged exposure to death, whereas the search for meaning played a less prominent role in psychological well-being [17]. Likewise, among palliative care nurses, participation in a meaning-centered intervention, consisting of guided reflection, experiential exercises, and group discussions based on Viktor Frankl's logotherapy, was linked to lower psychological distress, reduced burnout, and fewer negative emotions, while also enhancing positive emotions. However, actively seeking meaning in life appeared to correlate primarily with increased negative emotions [18].

Workplace conditions also play a crucial role in nurses' well-being and their risk of developing burnout [19]. The Job Demands–Resources (JD-R) model provides a theoretical framework for understanding how the balance between job demands and job resources influences occupational well-being, with excessive demands increasing the risk of burnout and adequate resources serving as protective factors [20]. A recent integrative review underscores that among nurses, specific job resources such as supervisor support, authentic leadership, interpersonal relationships, and autonomy consistently emerge as key determinants of well-being, engagement, and retention [21]. These resources not only buffer the negative impact of high job demands but also promote resilience and work satisfaction [22]. While much of the existing research focuses on job demands, less attention has been given to how job resources interact with personal factors, such as meaning in life, to influence burnout outcomes.

Building on this perspective, the present study examines the relationship between meaning in life (presence and search) and burnout symptoms among early- and mid-career nurses. We approach meaning in life as a domain-general resource, while explicitly situating its relevance in occupational contexts where burnout emerges. We specifically assess whether the presence of meaning is negatively associated with burnout symptoms, given its potential supportive role in occupational well-being. In contrast, an active search for meaning is hypothesized to be positively associated with burnout symptoms, as it may reflect existential uncertainty or psychological distress in demanding work environments. Additionally, we examine whether the following job resources—supervisor and colleague support (i.e., the perceived availability of emotional, instrumental, and performance-related support from one's immediate superior or peers), influence at work (i.e., perceived autonomy and control over various aspects of one's job), and opportunities for professional development (i.e., the extent to which one's job enables learning, growth, and the application or enhancement of skills)—moderate the relationship between meaning in life and burnout symptoms. Furthermore, we assess whether demographic and occupational characteristics such as age, gender, work experience, and professional function act as potential confounders. By doing so, this study offers insights into how workplace conditions may attenuate or exacerbate the associations between meaning in life and burnout symptoms among nurses. These findings can inform targeted organizational strategies aimed at fostering meaning, resilience and psychological well-being among early- and mid-career hospital nurses. Ultimately, the goal is to contribute to burnout prevention literature in healthcare settings.

## 2. Methods

### 2.1. Study Design

This study employed a cross-sectional design as part of a larger longitudinal mixed-methods panel study investigating the development of burnout symptoms among early- and mid-career hospital nurses. Baseline data collected between March and April 2024 were used to examine the relationship between meaning in life and burnout symptom scores. The analysis also accounted for psychosocial resources, demographic variables, and occupational factors. Ethical approval was granted by the Medical Ethics Committee of Ghent University Hospital (project number B6702024000168), and participant confidentiality was strictly maintained throughout the research process.

### 2.2. Setting and Participants

This study was conducted in six hospitals in Flanders, Belgium, including two university hospitals and four general hospitals. Eligible participants were nurses with up to 10 years of professional experience, encompassing HBO-5 nurses (associate degree nurses), professional bachelor nurses (registered nurses with a bachelor's degree in nursing), nurse specialists, deputy head nurses, and head nurses. Of the 20 hospitals invited to participate—representing an estimated eligible population of 100–500 nurses per general hospital and 800–1000 nurses per university hospital—nineteen responded. However, only six agreed to take part, citing workload pressures, frequent survey requests, and organizational restructuring as barriers to participation. Recruitment was facilitated by hospital HR and management teams, who disseminated study invitations and an online survey (via Qualtrics) through work email. The survey was distributed to approximately 2150 nurses, of whom 544 initiated or completed it. Following data cleaning, 375 participants were included in the final analysis, yielding an estimated response rate of 17.5%. Efforts to enhance participation included two reminder emails, assurances of anonymity, and hospital-specific feedback reports.

### 2.3. Variables and Measurements

#### 2.3.1. Primary Outcome: Burnout

The primary outcome variable in this study was burnout, measured using the Dutch version of the Burnout Assessment Tool (BAT) [23]. The BAT consists of 23 items, of which 12 assess the core symptoms of burnout across four subdimensions: emotional exhaustion (e.g., “At work, I feel mentally exhausted”), mental distance (e.g., “I feel a strong aversion to my work”), cognitive impairment (e.g., “At work, I have difficulty concentrating”), and emotional impairment (e.g., “I feel I have no control over my emotions at work”). Each subscale contains three items. Respondents indicated how frequently they experienced each symptom on a five-point Likert scale ranging from 1 (Never) to 5 (Always). A total burnout score was calculated by averaging all 12 core items, with higher scores reflecting greater burnout severity. The BAT has demonstrated robust psychometric properties in Dutch-speaking populations. The internal consistency of the total BAT core symptoms scale in the current sample was excellent (Cronbach's *α* = 0.87). The four subscales also demonstrated acceptable to high internal consistency: exhaustion (*α* = 0.79), mental distance (*α* = 0.66), cognitive dysregulation (*α* = 0.82), and emotional dysregulation (*α* = 0.83).

#### 2.3.2. Primary Exposure: Meaning in Life

The primary exposure, meaning in life, was assessed using the Meaning in Life Questionnaire (MLQ), a 10-item self-report instrument designed to measure both the presence of and the search for meaning, with five items per subscale [24]. The *presence of meaning* subscale reflects the extent to which individuals experience their lives as meaningful (e.g., “I understand my life's meaning”), whereas the *search for meaning* subscale captures the degree to which individuals are actively seeking meaning and purpose in life (e.g., “I am looking for something that makes my life feel meaningful”). All items were rated on a seven-point Likert scale ranging from 1 (absolutely untrue) to 7 (absolutely true). Subscale scores were computed by averaging the respective items, with higher scores indicating a greater presence or search for meaning. In the present study, the MLQ demonstrated good internal consistency. Cronbach's alpha coefficients were 0.86 for both the Presence of Meaning and Search for Meaning subscales, and 0.73 for the total scale.

#### 2.3.3. Moderators: Psychosocial Job Resources

Psychosocial job resources were measured using selected subscales from the validated Copenhagen Psychosocial Questionnaire (COPSOQ III) [25]. Four domains were included: supervisor support (2 items; e.g., “How often do you get help and support from your immediate superior, if needed?”), colleague support (2 items; e.g., “How often are your colleagues willing to listen to your problems at work, if needed?”), influence at work (4 items; e.g., “Do you have any influence on how you do your work?”), and possibilities for development (3 items; e.g., “Do you have the possibility of learning new things through your work?”). Items were rated on a five-point Likert scale ranging from 1 (never/to a very small extent) to 5 (always/to a very large extent). Mean scores were calculated for each subscale, with higher scores indicating more favorable perceptions of the psychosocial work environment. The four COPSOQ subscales included in the current study demonstrated good internal consistency. Cronbach's alpha coefficients were 0.89 for social support from supervisors, 0.91 for social support from colleagues, 0.81 for possibilities for development, and 0.71 for influence at work.

#### 2.3.4. Covariates: Demographic and Occupational Characteristics

Participants also reported demographic and occupational characteristics, including gender (male or female), age (21–25, 26–30, 31–35, 36–40, or 40+ years), professional function (registered nurse or associate degree nurse), and years of work experience (0–5 y, 5–10 years). These variables were included as covariates, as they were hypothesized to act as potential confounders in the relationship between meaning in life and burnout symptoms. Additional participant characteristics—such as employment percentage, living situation, level of education, institution type, and department of employment—were collected to provide a more detailed description of the study sample. These variables were used for descriptive purposes only and were not included in the main statistical analyses.

### 2.4. Statistical Methods

Descriptive statistics were used to summarize the data. Frequencies and percentages were reported for categorical variables. Normality was assessed using histograms and Q–Q plots, confirming that no transformations were required. Group differences in burnout symptoms across categorical variables were tested using *t*-tests or one-way ANOVAs. Bivariate associations among continuous variables (e.g., meaning in life, burnout subdimensions, and psychosocial job resources) were explored using Pearson correlation coefficients.

To examine the primary associations of interest, simple linear regressions were conducted to assess the independent effects of the two meaning in life subscales (presence and search) on all burnout symptom dimensions. A multiple linear regression model was then constructed including both subscales to evaluate their combined contribution. Potential confounding effects were examined by introducing demographic and occupational characteristics—including gender, age category, professional function, and years of work experience—into the multiple regression model. Variables were considered as confounders if the regression coefficient changed by at least 10%.

Subsequently, psychosocial job resources—social support from supervisors and colleagues, influence at work, and possibilities for development—were tested as potential moderators in the relationship between meaning in life and burnout symptoms. Interaction terms were included to explore potential moderation effects. All continuous variables were mean-centered prior to creating interaction terms, and categorical variables were dummy-coded. All statistical assumptions (linearity, homoscedasticity, independence, and normality) were verified. A *p* value < 0.05 was considered statistically significant. For interaction effects specifically, a threshold of *p* < 0.10 was applied to identify potential moderation effects. Significant interactions were further examined using split file analyses, comparing high and low groups based on median scores. Analyses were conducted using IBM SPSS Statistics version 29.

## 3. Results

### 3.1. Participants (Table 1)

The final sample consisted of 375 nurses, predominantly women (85.3%), with the majority aged 26–35 years (57.6%). Most participants held a Professional Bachelor's degree in Nursing (58.2%) and worked in general hospitals (57.3%), primarily in general nursing wards (61.8%). The gender and age distribution of our sample is broadly consistent with national statistics on the Belgian nursing workforce, which is predominantly female (85%–90%) and concentrated in younger age groups during the early stages of their careers [26].

### 3.2. Main Results

#### 3.2.1. Correlation Analysis: Preliminary Results (Table 2)

Pearson correlation analyses indicated that presence of meaning was significantly negatively correlated with the BAT total score (*r* = −0.357, *p* < 0.001) and all its subscales. In contrast, search for meaning was positively correlated with the BAT total score (*r* = 0.121, *p*=0.022) and with the exhaustion and mental distancing subscales, but not with emotional or cognitive dysregulation. Additionally, job resources were significantly negatively associated with burnout symptoms scores and positively correlated with presence of meaning, whereas search for meaning was not significantly related to these resources.

### 3.3. Associations Between Meaning in Life and Burnout Symptoms

#### 3.3.1. Simple Linear Regressions

**Figure 1:**
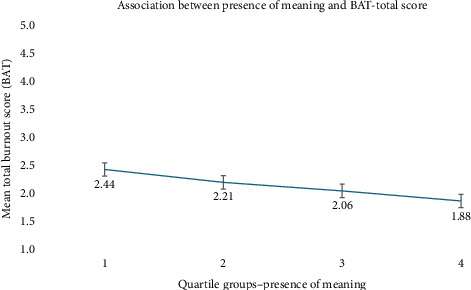
Mean burnout score (BAT total score) across quartiles of presence for meaning (MLQ). Error bars represent 95% confidence intervals.

**Figure 2:**
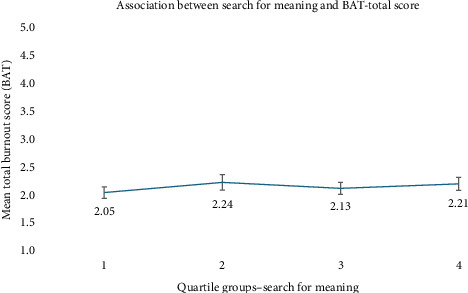
Mean burnout score (BAT total score) across quartiles of search for meaning (MLQ). Error bars represent 95% confidence intervals.

Simple linear regression models examined the relationship between meaning in life and burnout symptoms (Table 3). Presence of Meaning was significantly negatively associated with total burnout (*B* = −0.037, 95% CI [–0.047, −0.027], *p* < 0.001) and other subdimensions, including exhaustion, mental distancing, cognitive dysregulation, and emotional dysregulation (all *p* < 0.001). In contrast, Search for Meaning was positively associated with total burnout (*B* = 0.011, 95% CI [0.002, 0.021], *p*=0.022), as well as with the subdimensions exhaustion and mental distancing. No significant associations were found for the subdimensions cognitive dysregulation and emotional dysregulation. Figures 1 and 2 show the associations between meaning in life and total burnout symptoms across four quartiles.  Figure 1 confirms a negative linear relationship, with higher presence of meaning linked to lower burnout.  Figure 2 shows no clear linear trend for search for meaning; mean burnout scores fluctuate slightly across quartiles, with modest increases in the second and fourth quartiles.

#### 3.3.2. Multiple Linear Regression: Presence and Search for Meaning Combined Without Covariates

We next analyzed a model that included both presence and search for meaning as independent variables, without adjusting for covariates, to examine their combined associations with burnout symptoms. In this model (Table 3), presence of meaning remained significantly associated with lower overall burnout (*B* = −0.036, 95% CI [–0.047, −0.026], *p* < 0.001), whereas the association with search for meaning was no longer statistically significant (*B* = 0.008, 95% CI [–0.017, 0.001], *p*=0.082). For the subdimension exhaustion, both presence (*B* = −0.046, *p* < 0.001) and search (*B* = 0.017, *p*=0.010) were significantly associated. Across the remaining BAT subdimensions, presence of meaning consistently showed significant negative associations (all *p* < 0.001), while search for meaning was not significantly associated with any other dimension.

#### 3.3.3. Multiple Linear Regression: Examining Confounders and Interaction Effects

##### 3.3.3.1. Adjusting for Potential Confounders: Demographic and Occupational Characteristics

To assess whether associations between meaning in life and burnout symptoms remained after accounting for potential confounding variables, multiple linear regression analyses were conducted, adjusting for gender (male, female), age category (18–25 y, 26–30 y, 31–35 y, 36–40 y, 40+ y), work experience (0–5 years, 5–10 years), and professional function (professional bachelor nurse, associate degree nurse). As presented in Table 3, the inclusion of these demographic and occupational variables resulted in only minor changes in the coefficients for both subscales, with all changes remaining below the 10% threshold. Therefore, these variables did not meet the criterion to be considered confounders. Presence of Meaning remained consistently and significantly associated with lower levels of total burnout (*B* = −0.039, 95% CI [–0.049, −0.029], *p* < 0.001), as well as with each individual burnout dimension, including exhaustion, mental distancing, cognitive dysregulation, and emotional dysregulation. For Search for Meaning, associations with burnout symptoms remained generally weak. A small but statistically significant association with total burnout was observed in the fully adjusted model (*B* = 0.009, 95% CI [–0.000, 0.017], *p*=0.045), while significant associations were also found for exhaustion (*B* = 0.019, 95% CI [0.006, 0.031], *p*=0.005) and mental distancing (*B* = 0.012, 95% CI [–0.001, 0.024], *p*=0.037). No significant associations were found for cognitive or emotional dysregulation across any models.

##### 3.3.3.2. Moderation by Job Resources: Testing Interaction Effects

**Figure 3:**
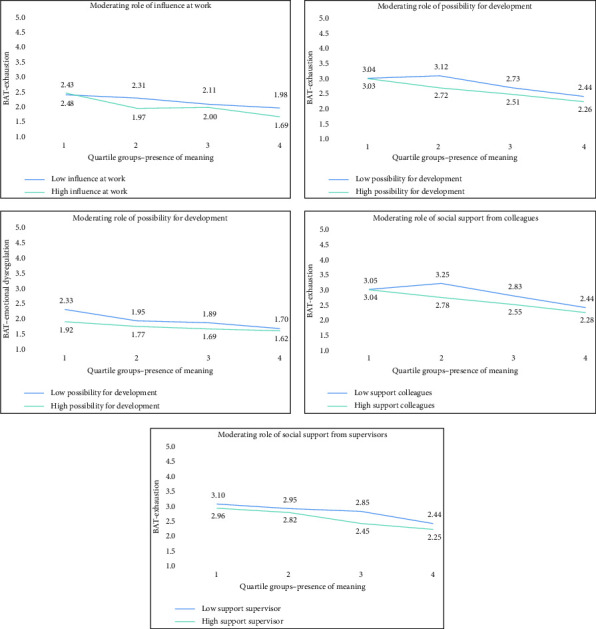
Graphs representing moderating effects of social support from colleagues and supervisors, opportunities for development and influence at work, on the relationship between presence of meaning and various burnout dimensions (exhaustion and emotional dysregulation).

**Figure 4:**
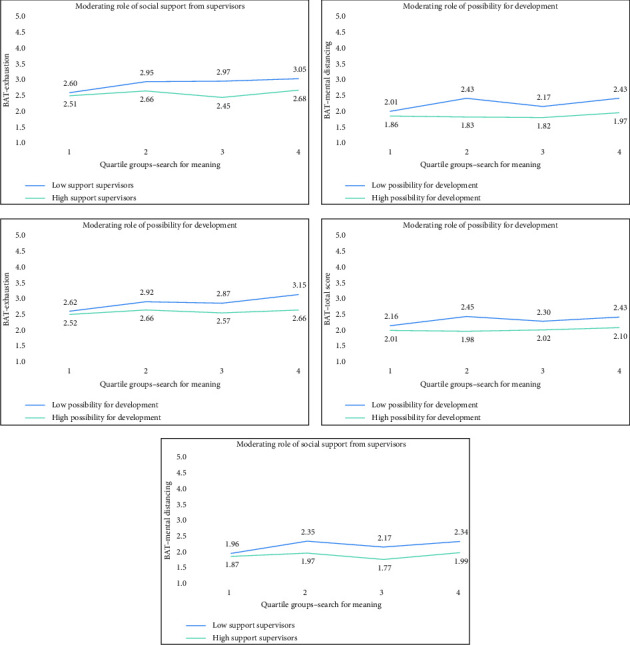
Moderating effects of social support from supervisors and opportunities for development on the relationship between search for meaning and various burnout dimensions (BAT total score, dysregulation, exhaustion and mental distancing).

To examine whether job resources moderate the relationship between meaning in life and burnout symptoms, interaction terms were tested (Appendix Tables A1 and A2), and significant interactions were further explored using median-split analyses (Figures 3 and 4, Appendix Tables A3 and A4). In these analyses, ‘high' and ‘low' job resources reflect groups split at the median of each resource variable. Nurses in the ‘high resource' group thus reported relatively favorable working conditions, whereas those in the ‘low resource' group reported comparatively fewer resources. The figures illustrate how these contextual differences shape the associations between meaning in life and burnout symptoms. For presence of meaning, its negative association with exhaustion was stronger for individuals with high job resources: influence at work (*B* = −0.067 vs. −0.037, both *p* < 0.001), development opportunities (*B* = −0.050, *p* < 0.001 vs. −0.034, *p*=0.006), and supervisor support (*B* = −0.057, *p* < 0.001 vs. −0.028, *p*=0.021). Colleague support was only significant in the high-support group (*B* = −0.054, *p* < 0.001). For emotional dysregulation, the negative association for presence of meaning remained in both groups but had a greater effect when development opportunities were low (*B* = −0.043, *p* < 0.001 vs. *B* = −0.017, *p*=0.039). For search for meaning, its positive association with exhaustion and mental distancing was significant only in the low development opportunities group (exhaustion: *B* = 0.036, *p*=0.002; mental distancing: *B* = 0.028, *p*=0.015), but not in the high group. Similarly, for supervisor support, search for meaning was significantly associated with higher exhaustion (*B* = 0.032, *p*=0.004) and mental distancing (*B* = 0.024, *p*=0.021) only in the low support group, but not when support was high. Colleague support did not moderate the relationship with emotional dysregulation.

## 4. Discussion

Our findings highlight distinct associations between presence and search for meaning in relation to burnout symptoms, underscoring their different roles in occupational well-being. Presence of meaning was consistently and negatively associated with all BAT dimensions, even after accounting for demographic and occupational characteristics. This suggests that individuals with a stronger sense of meaning could potentially report lower burnout symptoms, regardless of the workplace factors tested in this study. In contrast, search for meaning was positively associated with burnout symptoms, particularly exhaustion, and remained significant for total BAT scores after controlling for confounders, although the effect was small. This finding indicates that ongoing existential searching could potentially be associated to higher burnout symptoms.

Our results align with existing literature that distinguishes between the presence and search for meaning in life. Presence of meaning, experiencing life as coherent, purposeful, and significant, has consistently been associated with psychological well-being, resilience, and lower levels of distress [11, 14]. In contrast, the search for meaning showed a more complex pattern. Research suggests that searching without a sense of resolution can signal identity diffusion or existential insecurity, particularly in high-stress professions like nursing [27]. Shin and Steger emphasized that while the search for meaning can foster growth, it may also indicate a deficit state, especially when individuals lack resources or guidance to support this process [15]. In our study, presence and the search for meaning were not significantly correlated (*r* = −0.099), suggesting that they are distinct constructs rather than opposing ends of a single continuum. This finding reinforces the importance of examining both dimensions separately when exploring their implications for psychological outcomes such as burnout [24]. Importantly, our analyses showed that search for meaning was most strongly associated with burnout symptoms in low-resource environments. Drawing on Frankl's logotherapy, this existential searching may result in frustration when unaccompanied by sufficient resources, but in supportive contexts it may evolve into a constructive, growth-oriented process [28]. This underscores the dual nature of the search for meaning as both a potential vulnerability and a source of development, depending on contextual conditions.

The JD-R model helps further explain our associations. This model posits that job demands contribute to burnout, while job resources buffer against occupational stress and promote well-being [20]. While meaning in life is not explicitly part of the JD-R model, our findings suggest that its relationship with burnout may be influenced by the availability of job resources. Specifically, high job resources amplified the negative association between presence of meaning and burnout symptoms. Conversely, high job resources mitigated the distress associated with searching for meaning. The positive association between searching for meaning and burnout symptoms was strongest among nurses with fewer resources but became non-significant in the high-resource group. This suggests that in resource-rich settings, the negative effects associated with searching for meaning may weaken due to enhanced support and coping mechanisms. Furthermore, a stronger sense of meaning in life can be considered a personal resource: a psychological asset that enhances resilience, motivation, and well-being. Within the JD-R framework, personal resources can foster self-efficacy, optimism, and intrinsic motivation [29]. This supports individuals in navigating challenges more constructively and sustaining engagement despite job demands. These results underscore the need for structured job resources in healthcare professions, such as clear career development (possibilities for development), supportive and positive mentorship (social support), and engagement strategies to mitigate burnout (influence at work) [30, 31]. This is particularly important for early-career nurses, who are still developing and navigating their professional identity [6, 7]. Our findings suggest that access to job resources and a strong sense of meaning in life may contribute to this identity-forming process by fostering a sense of purpose, clarity, and resilience in their evolving professional roles.

Beyond situational context, meaning in life is not a static concept but a dynamic and evolving construct shaped by life stage, psychological development, and biographical events. Younger individuals, such as early-career nurses, may be more actively engaged in searching for meaning as part of identity formation, whereas presence of meaning may become more consolidated later in life [32]. In our study, meaning in life was examined as a cross-sectional construct. Although we included age categories (18–25, 26–30, 31–35, 36–40, 40+) and professional experience (0–5 vs. 5–10 years) as covariates, we recognize that these variables do not fully reflect participants' psychological life stages. For example, a nurse could begin working at age 22 or at 35, meaning that years of experience alone do not capture where someone is in terms of personal development or identity formation. To explore this further, we conducted additional analyses to test whether age group (< 30 vs. ≥ 30 years) and experience group (0–5 vs. 5–10 years) moderated the relationship between meaning in life and burnout symptoms. These analyses showed no significant interaction effects, suggesting that the associations between meaning in life and burnout were consistent across both younger and older participants, as well as between early- and mid-career nurses (results not shown).

This study has several strengths. Focusing on early- and mid-career nurses addresses a critical gap in occupational well-being research, as this group is particularly vulnerable during the transition from education to professional practice. Additionally, the statistical approach, which adjusted for job-related confounders and tested moderation effects, offers a nuanced understanding of how meaning in life interacts with workplace conditions. The use of validated instruments—BAT, MLQ, and COPSOQ—ensures reliable measurement, facilitating comparison with broader research on burnout and meaning [23–25].

However, several limitations should be considered. First, the low response rate (17.5%) raises concerns about potential selection bias. Unfortunately, we were unable to compare respondents with non-respondents, as no data were available for the latter. This limitation implies that nurses who chose to participate may differ systematically from those who did not, for example, in their levels of well-being or motivation. Nurses experiencing severe burnout symptoms may have been less likely to participate, potentially underestimating burnout severity in our current study. The relatively high attrition rate (31.1%) may have introduced additional bias. Furthermore, self-reported measures may be subject to recall or social desirability bias, potentially affecting the accuracy of responses.

Second, the cross-sectional design precludes causal inference. It remains unclear whether a strong sense of meaning reduces burnout symptoms, or whether lower burnout facilitates a stronger sense of meaning. Although we conducted additional analyses to explore whether age and professional experience moderated this relationship, longitudinal research is needed to better understand how meaning in life evolves and relates to burnout across different age groups and career stages. Qualitative approaches may also help capture biographical processes in more detail, explaining how different life phases and key career transitions influence the complex relationship between meaning in life and occupational well-being.

A methodological limitation is the high number of statistical tests conducted across multiple regression models, burnout subdimensions, and interaction terms. This increases the risk of type I errors (false positives) in the absence of corrections for multiple comparisons. In line with Rothman (1990), we chose not to apply formal corrections, as our hypotheses were theory-driven and such corrections may increase type II errors (false negatives), potentially obscuring meaningful relationships in exploratory research [33]. This rationale aligns with arguments that strict correction procedures may be overly conservative, particularly in health sciences, where identifying potential effects is valuable for guiding future research [34]. Nonetheless, this approach introduces the possibility that some marginally significant findings (e.g., the association between search for meaning and total burnout, *B* = 0.009, *p*=0.045; or mental distancing, *B* = 0.012, *p*=0.037), may reflect chance findings rather than true effects.

A fourth limitation concerns external validity. The study focused exclusively on nurses working in hospitals in Flanders, Belgium. The Belgian healthcare system, with its specific nursing roles, workload structures, and organizational policies, may not be representative of other settings, such as primary care, home care, or nursing homes, or of systems in other countries with different structural or cultural characteristics.

Finally, intervention studies are needed to test strategies aimed at fostering meaning in life and mitigating burnout. Programs such as meaning-centered training (e.g., focused on reflection and value clarification) or mentorship-based interventions that connect early-career nurses with experienced role models could help strengthen the presence of meaning or guide the search for meaning in a constructive way [18, 31, 35]. Practical approaches in hospital settings, such as reflective supervision or peer support groups, may also support nurses in cultivating meaning and reducing the risk of burnout [36].

## 5. Conclusion

In conclusion, this study offers insights into the role of meaning in life in relation to burnout symptoms among early- and mid-career nurses. By differentiating between the presence of meaning and the search for meaning, we highlight the distinct ways these two dimensions relate to occupational well-being. The findings suggest that fostering a strong sense of meaning in life may play a role in mitigating burnout symptoms, whereas existential searching—especially in work environments with limited resources—may exacerbate them. Nevertheless, it is important to note that the cross-sectional nature of this study precludes causal inferences. Future longitudinal research is needed to determine the directionality of these associations and to establish whether enhancing meaning in life and job resources can indeed reduce burnout symptoms among nurses. These insights carry several practical implications for healthcare organizations and policymakers. Initiatives aimed at enhancing job resources—such as structured mentorship programs, clear career progression pathways, and opportunities for professional development—could help create environments that both buffer against burnout and support the development of meaning in work [30, 31]. Creating space to explore personal values, engage in peer support, and connect meaningfully with patient care, may be especially beneficial for early-career nurses and could strengthen resilience and intrinsic motivation [35]. Moreover, fostering a workplace culture that acknowledges the emotional and existential dimensions of nursing, rather than focusing solely on efficiency, may contribute to more sustainable and fulfilling nursing careers [37]. Such efforts may not only improve individual well-being, but also support retention, engagement, and care quality across healthcare systems.

## Figures and Tables

**Table 1:** Description of demographic and professional characteristics.

Variable (*n*)	Category	Percent (*N*)
Gender (375)	Male	14.7% (55)
	Female	85.3% (320)

Age category (375)	18–25	17.1% (64)
	26–30	33.6% (126)
	31–35	24% (90)
	36–40	9.9% (37)
	40+	15.4% (58)

Working experience (375)	0–5 years	47.5% (178)
	5–10 years	52.5% (197)

Employment percentage (375)	100%	60.8% (228)
	80%–99%	34.1% (128)
	< 80%	5.1% (19)

Living situation (374)	Single with our without children	28.9% (108)
	Living with partner without children	30.7% (115)
	Living with partner with children	40.4% (151)

Highest education (373)	Master's degree	13.9% (52)
	Professional bachelor in nursing	58.2% (217)
	HBO-5 (associate degree) in nursing	27.9% (104)

Function (369)	Leadership role	5.2% (19)
	Registered nurse (bachelor)	66.9% (247)
	Associate degree nurse (HBO-5)	27.9% (103)

Institution type (361)	University hospital	42.7% (154)
	General hospital	57.3% (207)

Department (361)	General care units	61.8% (223)
	Critical care units	27.4% (99)
	Administrative and support services	10.8% (39)

**Table 2:** Pearson correlations between burnout symptoms, meaning in life and workplace resources.

Variable	Presence of meaning	Search for meaning	Possibilities for development	Influence at work	Social support supervisors	Social support colleagues
BAT–total score	−0.36^∗∗∗^	0.12^∗^	−0.34^∗∗∗^	−0.21^∗∗∗^	−0.32^∗∗∗^	−0.26^∗∗∗^
BAT–exhaustion	−0.32^∗∗∗^	0.16^∗∗^	−0.20^∗∗∗^	−0.22^∗∗∗^	−0.30^∗∗^	−0.19^∗∗∗^
BAT–mental distancing	−0.30^∗∗∗^	0.11^∗^	−0.38^∗∗∗^	−0.21^∗∗∗^	−0.29^∗∗∗^	−0.29^∗∗∗^
BAT–emotional dysregulation	−0.24^∗∗∗^	0.02	−0.24^∗∗∗^	−0.15^∗∗∗^	−0.29^∗∗∗^	−0.18^∗∗∗^
BAT–cognitive dysregulation	−0.25^∗∗∗^	0.07	−0.24^∗∗∗^	−0.07	−0.11^∗^	−0.14^∗^
Presence of meaning	*NA*	−0.09	0.28^∗∗∗^	0.13^∗^	0.21^∗∗∗^	0.21^∗∗∗^
Search for meaning	−0.09	*NA*	0.01	0.05	−0.05	−0.03

*Note:* Pearson correlation coefficients (*r*) between burnout dimensions (BAT), meaning in life, and workplace resources. Negative correlations indicate that higher meaning and workplace resources are associated with lower burnout scores, and vice versa. Significant correlations: ^∗^< 0.05 ^∗∗^< 0.010 ^∗∗∗^< 0.001.

**Table 3:** Linear regression analyses examining the relationship between meaning in life and burnout dimensions, adjusting for demographic and occupational characteristics.

Dependent variables	Level of analysis^∗^	Presence of meaning (B, CI, ^∗^*p*)	Search for meaning (B, CI, ^∗^*p*)
BAT–total	1	−0.04 [−0.05, −0.03]^∗∗∗^	0.01 [0.00, 0.02]^∗^
	2	−0.04 [−0.05, −0.03]^∗∗∗^	0.01[−0.00, −0.02]
	3	−0.04 [−0.05, −0.03]^∗∗∗^	0.01 [−0.00, 0.02]^∗^

BAT–exhaustion	1	−0.05 [−0.06, −0.03]^∗∗∗^	0.02 [0.01, −0.03]^∗∗^
	2	−0.05 [−0.06, −0.03]^∗∗∗^	0.02 [0.00, 0.03]^∗^
	3	−0.05 [−0.06, −0.03]^∗∗∗^	0.02 [0.01, 0.03]^∗∗^

BAT–mental distancing	1	−0.04 [−0.06, −0.03]^∗∗∗^	0.01 [0.00, 0.03]^∗^
	2	−0.04 [−0.05, −0.03]^∗∗∗^	0.01 [−0.00, 0.02]
	3	−0.05 [−0.06, −0.03]^∗∗∗^	0.01 [−0.00, 0.02]^∗^

BAT–cognitive dysregulation	1	−0.03 [−0.04, −0.02]^∗∗∗^	0.01 [−0.00, 0.02]
	2	−0.03 [−0.04, −0.02]^∗∗∗^	0.01 [−0.00, 0.01]
	3	−0.03 [−0.04, −0.02]^∗∗∗^	0.01 [−0.00, 0.01]

BAT–emotional dysregulation	1	−0.03 [−0.05, −0.02]^∗∗∗^	0.00 [−0.01, 0.02]
	2	−0.03 [−0.05, −0.02]^∗∗∗^	0.00 [−0.01, 0.02]
	3	−0.04 [−0.05, −0.02]^∗∗∗^	0.00 [−0.01, 0.01]

*Note:* Level of analysis^∗^: (1) Simple linear regression: Presence of meaning and search for meaning analysed separately. (2) Multiple linear regression: Presence of meaning and search for meaning combined without covariates. (3) Multiple linear regression with confounders: Presence of meaning and search for meaning combined, adjusted for confounding effects for demographic and occupational characteristics (gender, age category, work experience and professional function). Significant correlations: ^∗^< 0.05 ^∗∗^< 0.010 ^∗∗∗^< 0.001.

**Table 4:** Interaction effects between presence of meaning and job resources on BAT dimensions.

Dependent variables	Presence × influence at work^∗^	Presence × possibility for development^∗^	Presence × social support supervisors^∗^	Presence × social support colleagues^∗^
BAT–total	0.00 [−0.001, 0.000]	0.00 [0.000, 0.001]	0.00 [−0.001, 0.000]	0.00 [−0.001, 0.000]
BAT–exhaustion	−0.001 [−0.002, 0.000]^∗∗^	−0.001 [−0.001, 0.000]^∗^	−0.001 [−0.001, 0.000]^∗∗^	−0.001 [−0.002, 0.000]^∗^
BAT–mental distancing	0.00 [−0.001, 0.000]	0.00 [0.000, 0.001]	0.001 [−0.001, 0.000]	0.00 [−0.001, 0.000]
BAT–cognitive dysregulation	0.00 [−0.001, 0.000]	0.00 [0.000, 0.001]	0.00 [−0.001, 0.000]	0.00 [−0.001, 0.000]
BAT–emotional dysregulation	0.00 [−0.001, 0.001]	0.001 [0.000, 0.001]^∗^	0.00 [−0.001, 0.000]	0.00 [−0.001, 0.000]

*Note:* Each model included two main effects—Presence of meaning and one job resource (influence at work, possibility for development, supervisor support, or colleague support)—along with their interaction term. Significant correlations: ^∗^< 0.05 ^∗∗^< 0.010 ^∗∗∗^< 0.001.

^∗^(B, CI, *p*).

**Table 5:** Interaction effects between search for meaning and job resources on BAT dimensions.

Dependent variables	Search × influence at work^∗^	Search × possibility for development^∗^	Search × social support supervisors^∗^	Search × social support colleagues^∗^
BAT–total	0.00 [−0.001, 0.000]	3.084*E* − 5 [0.000, 0.000]^∗^	0.00 [−0.001, 0.000]	0.00 [0.000, 0.001]
BAT–exhaustion	0.00 [−0.001, 0.000]	5.622*E* − 5 [0.000, 0.000]^∗^	−0.001 [−0.001, 0.000]^∗^	0.00 [−0.001, 0.001]
BAT–mental distancing	0.00 [−0.001, 0.000]	0.00 [−0.001, 0.000]^∗^	0.00 [−0.001, 0.000]	−4.907*E* − 5 [−0.001, 0.001] 0.690
BAT–cognitive dysregulation	0.00 [−0.001, 0.000] 0.412	0.00 [0.000, 0.000] 0.372	0.00 [0.000, 0.001] 0.615	−0.004 [−0.008, −0.001] 0.431
BAT–emotional dysregulation	0.00 [−0.001, 0.000]	0.00 [0.000, 0.000]	0.00 [0.000, 0.001]	0.001 [0.000, 0.001]

*Note:* Each model included two main effects—Search for meaning and one job resource (influence at work, possibility for development, supervisor support, or colleague support)—along with their interaction term. Significant correlations: ^∗^< 0.05 ^∗∗^< 0.010 ^∗∗∗^< 0.001.

^∗^(B, CI, *p*).

**Table 6:** Unadjusted stratified regression analyses showing the association between presence of meaning and BAT dimensions at low and high levels of job resources.

Dependent variable		Influence at work^∗^	Possibility for development^∗^	Social support supervisor^∗^	Social support colleagues^∗^
Exhaustion	Low resource	−0.04 [−0.05, −0.02]^∗∗∗^	−0.03 [−0.06, −0.01]^∗∗^	−0.03 [−0.05, −0.00]^∗^	−0.02 [−0.05, 0.01],
	High resource	−0.07 [−0.10, −0.04]^∗∗∗^	−0.05 [−0.07, −0.03]^∗∗∗^	−0.06 [−0.08, −0.04]^∗∗∗^	−0.05 [−0.07, −0.04]^∗∗∗^

Emotional dysregulation	Low resource	NA	−0.04 [−0.07, −0.02]^∗∗∗^	NA	NA
	High resource	NA	−0.017 [−0.03, −0.00]^∗^	NA	NA

*Note:* Significant correlations: ^∗^< 0.05 ^∗∗^< 0.010 ^∗∗∗^< 0.001.

Abbreviation: NA = not applicable.

^∗^(B, CI, *p*).

**Table 7:** Unadjusted stratified regression analyses showing the association between search for meaning and BAT dimensions at low and high levels of job resources.

		Possibility for development^∗^	Social support supervisor^∗^	Social support colleagues^∗^
Total BAT score	Low resource	0.02 [−0.00, 0.03]^∗^	NA	NA
	High resource	0.01 [−0.00, 0.02]	NA	NA

Exhaustion	Low resource	0.04 [0.01, 0.06]^∗∗^	0.03 [0.01, 0.05]^∗∗^	NA
	High resource	0.01 [−0.00, 0.03]	0.01 [−0.002, 0.031]^∗^	NA

Mental distancing	Low resource	0.03 [0.01, 0.05]^∗^	0.02 [0.00, 0.04]^∗^	NA
	High resource	0.01 [−0.01, 0.02]	0.01 [−0.01, 0.02]	NA

Emotional dysregulation	NA	NA	NA	−0.02 [−0.05, 0.01]
	NA	NA	NA	0.01 [−0.01, 0.02]

Note: Significant correlations: ^∗^< 0.05 ^∗∗^< 0.010 ^∗∗∗^< 0.001.

Abbreviation: NA = Not Applicable.

^∗^(B, CI, *p*).

## Data Availability

The datasets generated and/or analyzed during the current study are not publicly available due to privacy concerns of the participants.

## Nomenclature

BAT: Burnout Assessment Tool
MLQ: Meaning in Life Questionnaire
COPSOQ: Copenhagen Psychosocial Questionnaire
JD-R: Job Demands–Resources
